# Dopamine, Salience, and Response Set Shifting in Prefrontal Cortex

**DOI:** 10.1093/cercor/bhu210

**Published:** 2014-09-21

**Authors:** T. Shiner, M. Symmonds, M. Guitart-Masip, S. M. Fleming, K. J. Friston, R. J. Dolan

**Affiliations:** 1 Wellcome Trust Centre for Neuroimaging, Institute of Neurology, University College London, London WC1N 3BG, UK; 2 Nuffield Department of Clinical Neurosciences, University of Oxford, John Radcliffe Hospital, Oxford OX3 9DU, UK; 3 Ageing Research Center, Karolinska Institute, SE-113 30Stockholm, Sweden; 4 Center for Neural Science, New York University, New York, NY 10003, USA; 5 Department of Experimental Psychology, University of Oxford, Oxford OX1 3UD, UK

**Keywords:** dopamine, fMRI, prefrontal cortex, reversal learning, set shifting

## Abstract

Dopamine is implicated in multiple functions, including motor execution, action learning for hedonically salient outcomes, maintenance, and switching of behavioral response set. Here, we used a novel within-subject psychopharmacological and combined functional neuroimaging paradigm, investigating the interaction between hedonic salience, dopamine, and response set shifting, distinct from effects on action learning or motor execution. We asked whether behavioral performance in response set shifting depends on the hedonic salience of reversal cues, by presenting these as null (neutral) or salient (monetary loss) outcomes. We observed marked effects of reversal cue salience on set-switching, with more efficient reversals following salient loss outcomes. l-Dopa degraded this discrimination, leading to inappropriate perseveration. Generic activation in thalamus, insula, and striatum preceded response set switches, with an opposite pattern in ventromedial prefrontal cortex (vmPFC). However, the behavioral effect of hedonic salience was reflected in differential vmPFC deactivation following salient relative to null reversal cues. l-Dopa reversed this pattern in vmPFC, suggesting that its behavioral effects are due to disruption of the stability and switching of firing patterns in prefrontal cortex. Our findings provide a potential neurobiological explanation for paradoxical phenomena, including maintenance of behavioral set despite negative outcomes, seen in impulse control disorders in Parkinson's disease.

## Introduction

Shifting from one pattern of behavioral response to a more appropriate action in the face of unexpected or surprising events is central to adaptive behavior. Dopamine is implicated in shifting response set, including triggering of a behavioral switch (Cools, Lewis et al. 2006; Lee et al. 2007), learning new associations (Shohamy et al. 2005), maintenance of learned associations (Cohen et al. 2002), commission of action (Frank et al. 2004), and both tracking and responding to rewarding or punishing (i.e., hedonic or valenced) outcomes in decision making (Cools et al. 2009; van der Schaaf et al. 2014). Several brain regions, all of which receive strong dopaminergic innervation, are thought to support this process, including medial and lateral prefrontal cortex as well as the striatum (Fellows and Farah 2003; Izquierdo et al. 2004; Robinson et al. 2010; Rygula et al. 2010; Hampshire et al. 2012; Fallon et al. 2013).

It has been suggested that aberrant attribution of salience to cues is central in obsessive–compulsive disorder, addiction and depression (Diekhof et al. 2008), as well as in the impulse control disorders observed in Parkinson's disease (Dagher and Robbins 2009). However, dopaminergic modulation has mixed effects on these processes. For example, in learning from negative feedback in patients with Parkinson's disease, dopamine can improve performance in some domains but exerts a detrimental effect on others (Cools et al. 2003; Frank et al. 2004; Shohamy et al. 2005, 2006; Shiner et al. 2012). Here, we sought to delineate the role of dopamine on the shifting of response set in healthy individuals, and specifically how this depends upon the hedonic salience (e.g., negative valence) of outcomes.

The neuronal dynamics encoding response set are thought to involve selection and maintenance within cortico-striatal circuits (Frank and Claus 2006; McNab and Klingberg 2008). Within this framework when expectations about outcomes are violated, as in a reversal, one pattern of neural activity is deselected allowing a new representation to emerge corresponding to the switch in response set (Deco and Rolls 2005; Friston 1997; Oullier and Kelso 2006; Rabinovich et al. 2008). Our working assumption is that an unexpected outcome, signifying a change in—and uncertainty about—contingencies, would cause a reduction in mesocortical dopaminergic modulation of prefrontal activity (Ferron et al. 1984), enabling the emergence of a new pattern of neuronal dynamics and response set. Furthermore, we predicted that a pharmacological boost to dopamine would preclude this reduction and compromise set-switching.

One standard view of dopamine—namely the reporting of reward prediction errors—is based on an assumption that subjects need to learn the value of subsequent actions (Schultz et al. 1997; Daw and Doya 2006; Niv and Schoenbaum 2008). Importantly, we employed a task in which stimulus–response–outcome mappings were deterministic (in contrast to most reversal paradigms where mappings are probabilistic), such that unexpected outcomes triggered a switch in response set rather than new learning, which is also dopamine-dependent. In this context when subjects have already learned response contingencies, reinforcement learning about actions has limited utility. Instead, subjects have to infer changes in contingencies before selecting the appropriate set. In this setting, dopamine may have a complimentary role in reporting the precision or confidence in beliefs about the consequences of action under the current set (Galea et al. 2012).

Unexpected outcomes following an action engender a striatal signal, reflecting a prediction error thought to emanate from the nigrostriatal dopamine system. Here, we predicted a corresponding reduction in prefrontal responses, due to uncertainty about current set, would be modulated by mesocortical dopaminergic inputs (Friston et al. 2012). This concurs with increases in striatal blood oxygenation level-dependent (BOLD) signal seen following salient outcomes such as reward omission (Pagnoni et al. 2002), or negatively valenced events such as loss or punishment on the one hand (Cools et al. 2002; Seymour et al. 2005) and, on the other hand, phasic decrements in BOLD signal following the unexpected absence of a reward in the context of a well-established stimulus–outcome train, in dorsal (Ramnani et al. 2004) and ventral (O'Doherty et al. 2003) regions of medial prefrontal cortex. Moreover, we asked whether prefrontal responses to unexpected outcomes would similarly depend upon the hedonic salience of outcome cues.

Previous findings from our laboratory have highlighted functional asymmetries in probabilistic learning requiring commission or omission of responses, respectively (Guitart-Masip, Huys et al. 2012), and widespread regions of prefrontal cortex are differentially activated by action versus action inhibition (Rubia et al. 2001; Criaud and Boulinguez 2013). Note here we were interested in distinguishing dopaminergic effects on set-switching separate from its effects on action execution. To control for differences in movement, we alternated between “go” and “no-go” response sets which enabled us to average over behavioral and physiological responses that did, and did not, involve executive motor components (and nonspecific behavioral inhibition), thereby isolating set-switching per se.

In summary, under placebo, we predicted a reduction in prefrontal responses following salient reversal cues (correlating with a putative demodulation of an established pattern of neural activity), engendering a switch between patterns of behavioral responses. Under l-dopa, we predicted an impaired ability to switch behavior following a salient reversal cue, and a concurrent disruption to the normal pattern of prefrontal responses. By employing a paradigm which both controlled for the role of dopamine in learning (by using deterministic outcomes) and in action execution (by using a Go/No-Go design), we aimed to isolate and test the unique role of dopamine in response set selection under different levels of hedonic salience.

## Materials and Methods

### Participants

The study and its procedures were approved by a UCL Research Ethics Committee. All participants gave written informed consent. Only male participants were included to avoid menstrual cycle-dependent interactions between gonadal steroids and the dopaminergic system (Becker and Cha 1989; Dreher et al. 2007). Sixteen healthy men [age mean (SE) 23.8 (1.65)] completed the study. Two further subjects completed the experiment but were not included in the analysis (1 in the dopamine session and 1 in the placebo session) due to excessive drowsiness (and excessively low response rates) during the scanning session. No participants were taking concurrent medication and none had a history of neurological or psychiatric illness.

### Stimuli and Task

#### Task Design

**Figure 1.**
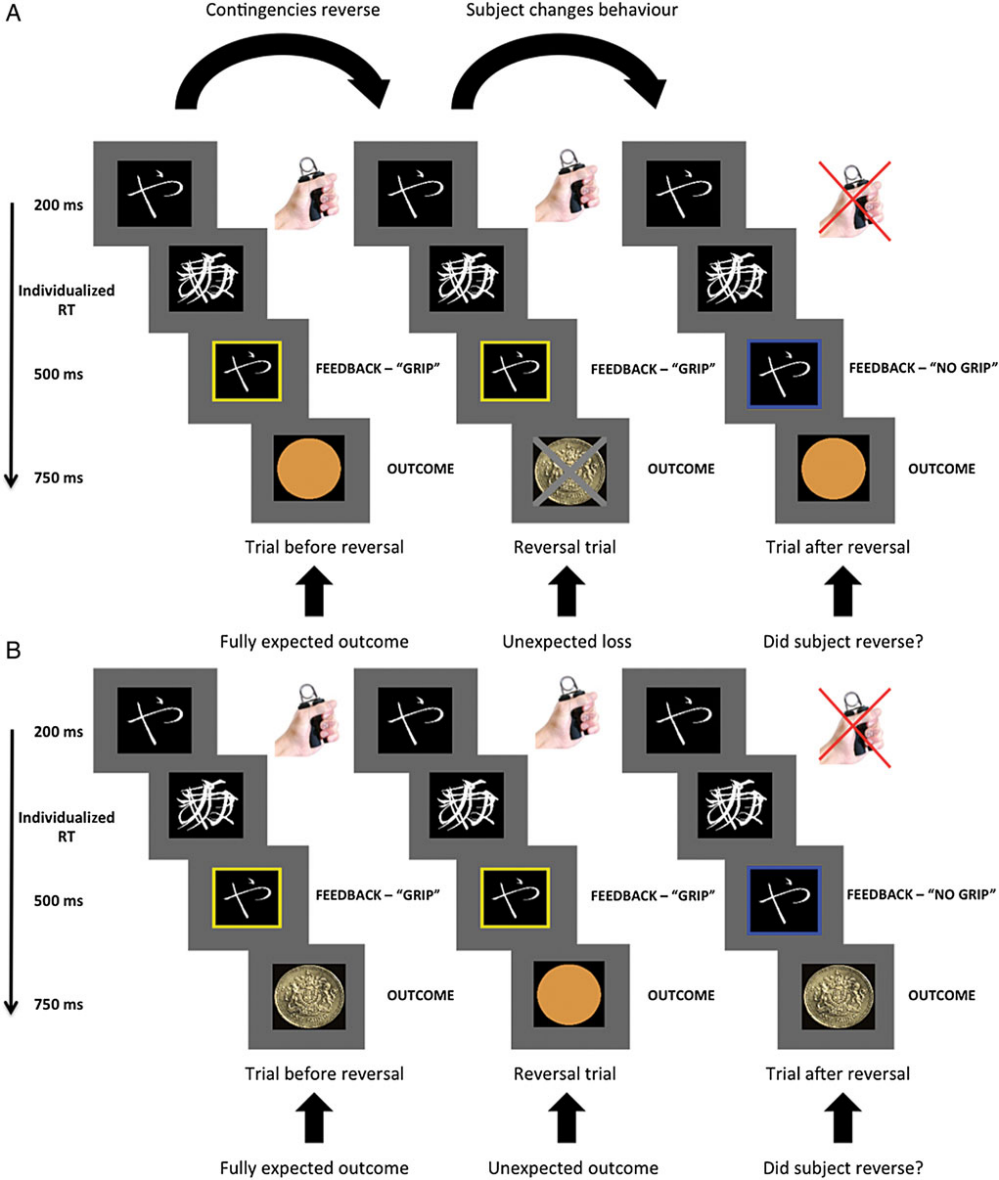
Task depiction. (*A*) Schematic of a reversal trial signaled by a salient monetary loss. Subjects have learnt the appropriate response to presentation of one of 2 paired symbols (Hiragana figures), either gripping (Go) or omitting a grip response (No-Go). Initially (left—trial before reversal, *T*_reversal − 1_) subjects observe a symbol signaling a grip is required; a yellow border then appears to indicate that the grip has been registered successfully. Subsequently, an outcome screen appears. This example is taken from a block where subjects avoid a monetary loss when performing correctly; hence, an empty circle is shown to inform that no money was lost. On the next trial (middle—reversal trial, *T*_reversal_), the contingencies have reversed and the elicited response of a grip is now incorrect, and the outcome screen signals loss of money (indicated by a pound sign with a cross through it). The alternative symbol (not shown) simultaneously switches its contingency from the complimentary “no-grip to avoid loss” to a “grip to avoid loss.” On the next trial (right—trial after reversal), the subject alters their behavioral response and now does not grip in response to the presentation of this symbol (indicated by the blue border). This is now the new correct response, and no money is lost (hence, the outcome screen shows an empty circle). (*B*) Schematic of a reversal trial signaled by a null outcome. In this example, subjects have learnt that the initial Hiragana symbol (left) signals that a grip is required, and a yellow border then appears to indicate that a grip has been registered successfully. In this block, the outcome screen indicates that they will receive money for a successful response (indicated by a pound coin). On the next trial (middle), the subject grips again; however, the contingencies have reversed and the elicited response of a grip is now incorrect. The outcome screen therefore signals a null outcome (indicated by an empty circle). On the next trial (right), the subject has changed behavior and now does not grip on observing the same symbol (indicated by the blue border) and hence receives money again for a successful response. In parallel, the alternative Hiragana symbol (not shown) will also switch contingencies on the reversal trial.

We used a novel reversal switching task (Fig. 1). Stimuli consisted of one of 4 Hiragana (Japanese font) symbols presented in white font on a black background. One pair of these symbols was presented in any 1 block (in pseudorandomized order). All trials followed the same sequence, with each block of trials lasting 17 min.

A block commenced with a single Hiragana symbol presented on-screen for 200 ms before being masked (by a composite of all 4 Hiragana symbol characters) for a period of time titrated to each subject's reaction time (RT) (see below). Subjects were then required to make an appropriate response, following presentation of one of the 2 symbols, either gripping (Go) or omitting a grip response (No-Go). In each block, one of the symbols instructed a grip and the other a no-grip response.

To provide feedback, a colored border then appeared (500 ms) around the unmasked Hiragana symbol, which indicated the registered response (Go; or No-Go) made on that trial; a yellow border for a grip (Go) and a blue border for no grip (No-Go). Following feedback subjects were then presented with an outcome screen (750 ms).

#### Reversals

At unpredictable intervals, the contingencies for the pair of symbols in each block were reversed (reversal shifts). Consequently, on reversal trials, a stimulus for which subjects had previously gripped (Go response) now instructed a no-grip response and vice versa. This meant that subjects performing the expected action would receive an unpredicted or surprising outcome—a signal to switch response set. To ensure that reversal shifts were unpredictable—and that subjects had established a specific response set prior to a reversal—we set a minimum constraint of 5 correctly executed trials (10 before the first reversal switch) per contingency before a possible reversal, after which there was a 50% probability of a reversal per trial. Reversals were balanced across motor responses (i.e., occurring equally on trials for expected “grip” and “no grip” responses).

#### “Null” and “Salient” Blocks

Reversals were cued in 2 different ways across block types. Six blocks were run in total (3 “null” and 3 “salient” blocks). In “null” blocks, reversals were signaled by neutral outcomes (signifying an incorrect response to the shifted contingency), while correct responses led to receipt of one pound (represented on-screen by an empty circle or a pound coin respectively). In “salient” blocks, reversals were signaled by loss of a pound (represented by a pound coin picture with a cross through it)—with no money being lost if responses were correct (shown as an empty circle on screen). Implicit here is the assumption that the visual presentation of a monetary loss represents a more hedonically salient event than the visual “null” events where expected monetary reward is absent, although both types of event are “salient” in the sense of being an unexpected outcome.

Real monetary rewards were provided (see reward schedule below). Critically, because reversals were rare events and contingencies were entirely deterministic, participants formed a strong expectation of performing correctly and receiving confirmatory feedback. In addition, by using fully deterministic outcomes and an appropriate time for response execution, we ensured minimal learning was required, thereby allowing us to focus on reversal shifts—as opposed to reinforcement learning about action contingencies which come into play within standard reversal tasks with probabilistic outcomes. Note that in describing these 2 block types, we focus on the outcomes signaling a reversal shift, as this is the behaviorally relevant trigger for a switch in response set. We measure behavioral performance as accuracy in the trial following a reversal (i.e., the trial following the unexpected outcome; *T*_reversal + 1_).

In the analysis of the neuroimaging data, we were specifically interested in neuronal responses on reversal shifts signaled by salient outcome cues, relative to neutral outcomes. We isolated these responses by comparing activation during reversal trials (*T*_reversal_), with an unexpected outcome, with the preceding trial (*T*_reversal − 1_) where outcomes are fully expected. The only difference between these consecutive trials is the signal (i.e., outcome cue) indicating a reversal shift and the subsequent activity we associate with set-switching.

### Procedure

#### Overview

We employed a within-subjects design. All subjects performed the task on placebo (500 mg soluble calcium, Cacit 1.25, Warner) or l-dopa (150 mg soluble levodopa, Madopar 187.5 mg, Roche). l-Dopa and placebo were dissolved in orange squash and both the participants and the investigator were blinded to the order of the drug/placebo. After ingestion, participants waited for 60 min to ensure maximum peak plasma drug concentration according to levodopa pharmacokinetics (Khor and Hsu 2007). The order was randomized and sessions were scheduled a minimum of 1 week apart—to ensure complete drug washout in cases where subjects had received drug on the first visit. Different Hiragana symbols were used for each visit (see Fig. 1). Participants performed both sessions at the same time of day to control for any diurnal fluctuations in baseline neurotransmitter levels.

#### Grip Measurement

To record motor responses, we used a pneumatic handgrip device held in the right hand (Kurniawan et al. 2010). This MRI-compatible device was molded from 2 plastic cylinders compressing an air tube connected to a transducer (Honeywell, Morristown, NJ, USA), which converted air pressure into a continuous voltage output. The signal was recorded (Spike2, Cambridge Electronic Design) and analyzed on-line in MATLAB 7.1 (www.mathworks.com). Individual maximal grip strength was measured and used to calibrate the threshold for recording a response (at 10% maximal grip). Visual stimuli were presented using Cogent 2000 (http://www.fil.ion.ucl.ac.uk/ and http://www.icn.ucl.ac.uk/) and Cogent Graphics (John Romoya, Wellcome Trust Centre for Neuroimaging, UCL).

#### Reaction Time Titration

We limited the time per trial that each subject had to emit a grip/no grip response. This ensured a train of prepotent responses (once contingencies had been learnt), such that only occasional errors were made (failure to switch response sets following a reversal trial). This RT limit was individually calibrated according to baseline grip RTs—allowing us to control for intrinsic within-subject variability in response speed. The baseline measurement, prior to drug/placebo administration, was setup similarly to the general trial sequence for the switch task. Two fractals were presented, one cueing a fast grip and one requiring omission of a grip response. These contingencies were explicitly described before the task, and feedback was given following each response. The average +2 SD of RT (of grip trials), averaged over 15 presentations, was used as the upper threshold time for a response in the reversal switch task. Average individualized RTs were 620 ms (SE: 35.2).

Subjects were fully informed of the deterministic contingencies, and that occasionally the mapping of symbols to their motor response would switch. To familiarize them with this task structure, they were first required in both sessions to undertake a short practice block prior to scanning. During the practice session, subjects performed the identical task, except with different Hiragana symbols. Each of 3 scanning runs, with concurrently collected behavioral data, lasted ∼17 min, and consisted of 2 randomized blocks, one in which the reversals were signaled by null outcomes and one in which the reversals were signaled by a “salient” monetary loss.

#### MRI Scanning

Imaging data were acquired using a 3T Siemens Allegra scanner equipped with a Siemens head coil. Anatomical images were acquired using magnetization-prepared rapid-acquisition gradient echo scans, which were followed by 1-mm-thick axial slices parallel to the anterior commissure–posterior commissure plane. Functional scans used a gradient echo sequence; repetition time, 2.86 s; echo time 25 ms; flip angle 90°; matrix size 128 × 72; field of view 192 mm; slice thickness, 2 mm. A total of 44 axial slices were sampled. The in-plane resolution was 3 × 3 mm.

#### Payment Schedule

Subjects received payment on completion of both sessions. To ensure incentive compatibility (i.e., so that subjects knew that each trial had the potential for real monetary loss or gain), 15 trials of each block type were randomly selected across sessions and paid out for real.

### Data Analysis

#### Behavior

The critical behavioral trials were the trials after a reversal shift signal (*T*_reversal + 1_). On the reversal trial (*T*_reversal_), unexpected feedback (reversal cues), either in the form of a more salient loss or a less salient null outcome, signaled the need to change behavior under 2 factors: Go versus No-Go and drug versus placebo. In other words, there were 4 trial types (Go to avoid loss, No-Go to avoid loss, Go to avoid null, No-Go to avoid null) executed under 2 drug states (placebo and l-dopa) in a fully balanced design. Accuracy (proportion correct responses on the trial following reversal, *T*_reversal + 1_) was computed for each trial type. Comparisons between trial types, at the group level, were performed using a three-way (reversal type × action × drug) repeated-measures ANOVA.

### Functional MRI

Functional imaging data were analyzed using statistical parametric mapping (SPM8; Wellcome Trust Centre for Neuroimaging, London, UK; http://www.fil.ion.ucl.ac.uk/spm). Images were realigned with the first volume (after discarding the first 6 dummy volumes), unwarped, normalized to the Montreal Neurological Institute reference brain, resampled to 3 × 3 × 3 mm^3^ voxels, and spatially smoothed (8-mm full-width at half-maximum).

We used a standard summary statistical approach to random-effects analyses at the between-subject level: at the within-subject level, we specified a general linear model (GLM) comprising regressors for each trial type. For the imaging analysis, we were specifically interested in the difference between feedback responses to the trial before reversal—with an expected outcome—and the reversal trial—with an unexpected reversal cue. We use this [*T*_reversal − 1_ − *T*_reversal_] difference to index brain activation preceding a response set shift, separately analyzing brain activation for salient versus null blocks. Trial-specific activations were modeled as box-car functions (with durations set according to trial length on an individual subject basis). Note this subsumes activity related to motor preparation, action, as well as responses to the outcome—by averaging over “go” and “no-go” response sets entailing behavioral and physiological responses that did, and did not, involve executive motor components (and nonspecific behavioral inhibition), we thereby isolate neural activity underlying response set-switching per se. The stimulus functions were then convolved with the canonical hemodynamic response function to form functional MRI (fMRI) regressors.

We focus on brain activation preceding behaviorally validated response set shifts; hence, we stratified trials into behaviorally correct and incorrect responses. Our design matrix therefore modeled effects in the following trial types: (correct) trial before reversal, *T*_reversal − 1_; (incorrect) reversal trial, *T*_reversal_ (note that this trial is by default incorrect because the contingencies have been changed experimentally); correct trial after reversal, *T*_reversal + 1_; incorrect trial after reversal *T*_reversal + 1_.We separately modeled all other correct and incorrect trials. Each block was modeled separately. The number of error trials (errors on trials excluding reversal and trial after reversal trials) was relatively small (<5%). Data from one subject had to be excluded due to a very high overall accuracy in the trial after reversal (>95%) thereby preventing our ability to contrast correct and incorrect responses to reversal trials.

Low-frequency drifts were removed with a high-pass filter (128-s cutoff). Short-term temporal autocorrelations were modeled using a two-parameter autoregression model. Motion correction regressors (from the realignment procedure) were entered as covariates of no interest. Statistical significance was assessed using linear contrasts of the regression coefficients from the GLM, generating statistical parametric maps (SPM) of *t*-values across the brain for each subject and contrasts of interest. Placebo and dopamine sessions were analyzed separately at the first (within-subject) level, and corresponding contrast images were taken to the second (between-subject) level for random-effects analysis. This entailed using contrasts for each subject in placebo and levodopa sessions in a group level GLM. Paired *t*-tests were used to produce SPMs testing for drug effects on responses to reversal cues.

Anatomical localization was implemented by overlaying t-maps on a normalized structural image (averaged across subjects). All coordinates are reported in MNI space. Statistical analyses to identify significant voxels were performed in SPM with whole brain family-wise error (FWE) correction at *P* = 0.05. Our primary regions of interest focused on principal dopaminergic targets, specifically striatum and prefrontal cortex including ventromedial prefrontal cortex (vmPFC) (Haber 2003); hence, we also report significant (FWE-corrected) voxels within these areas. To quantify (in-sample) effect sizes, we extracted response estimates from 4-mm spheres centered on peak voxels of activation (using MarsBAR) and show these averaged contrast estimates in separate bar plots.

## Results

### Behavior

To measure efficacy of reversal shifting, we calculated response accuracy (i.e., the proportion of correct responses) on the first trial postreversal.

**Figure 2.**
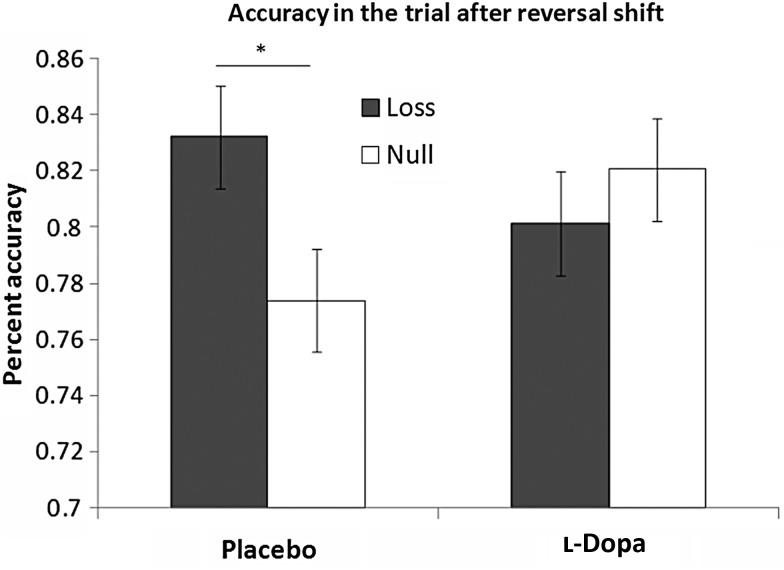
Accuracy trials after reversal shift. Accuracy (percentage correct responses) in the trial after a reversal shift for both reversal types (salient reversal cue—gray bars/null reversal cue outcome—white bars), and drug conditions (placebo and l-dopa). Bars show within-subject standard error. Under placebo, subjects are more accurate when reversing their behavior after a salient reversal cue compared with a neutral cue. When subjects were given l-dopa, this performance advantage was eliminated (*P < 0.05; see text for statistical reporting).

First, we characterized adaptive behavior under placebo and found subjects were significantly better at reversing after a salient reversal cue compared with a neutral cue (paired *t*-test, null vs. salient; *T*_(1,15)_ = −3.21, *P* = 0.006; Fig. 2). This supported our a priori prediction that a salient loss cue is a more potent catalyst for set-switching than an unexpected absence of a monetary gain.

We found a reversal type (salient/null) by drug (l-dopa/placebo) interaction (*F*_1,15_ = 11.73, *P* = 0.004) for performance accuracy in trials following a reversal. l-Dopa eliminated the performance advantage in trials signaled by a salient cue relative to a null cue. This resulted in accuracy under levodopa being equivalent for both conditions (l-dopa: salient vs. null, paired *t*-test; *t*_(1,15)_ = 1.0, *P* = 0.332; Fig. 2). Performance under null trials was worse in the placebo compared with the l-dopa group (*t*_(1,15)_ = −1.89, *P* = 0.078, two-tailed), albeit at trend significance.

Crucially, there was no main effect of drug (l-dopa/placebo) (*F*_1,15_ = 0.125, *P* = 0.728) or action (Go/No-Go) (*F*_1,15_ = 0.249, *P* = 0.625), on reversal accuracy. The absence of a drug effect on overall accuracy was surprising, and suggests levodopa administration leads to a selective blunting of set-switching following hedonically salient outcomes alone. There was no interaction between either valence (salient/null) and action (Go/No-Go) (*F*_1,15_ = 2.149, *P* = 0.163), or drug (l-dopa/placebo) and action (Go/No-Go) (*F*_1,15_ = 0.02, *P* = 0.888).

### fMRI

#### The Effect of l-Dopa on vmPFC Reversal Responses Depends on Salience

**Figure 3.**
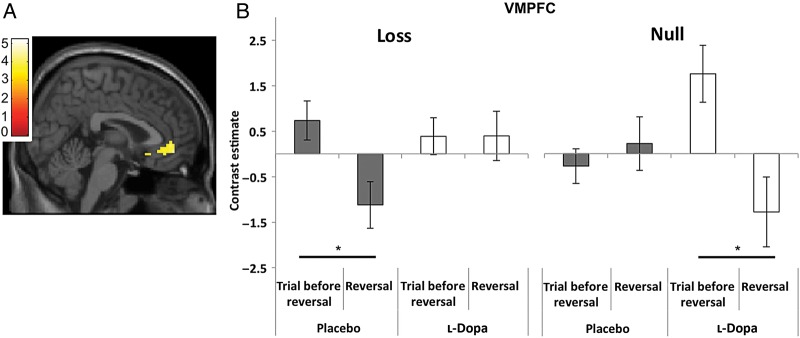
Neurophysiological responses to reversals in vmPFC. (*A*) Contrast of BOLD activation in the trial before a reversal (*T*_reversal − 1_, fully expected outcome) minus the reversal trial (*T*_reversal_, unexpected outcome) averaged over all conditions (placebo/l-dopa and salient/null). Trial-specific activations were modeled as box-car functions from trial onset, with durations set according to the trial length—as estimated on an individual subject basis. There was a significant reduction in vmPFC activation in response to a violation of expectations in reversal trials [Montreal Neurological Institute (MNI) space; peak (*x*, *y*, *z*) coordinates: −3, 11, −2; *z* = 3.84, *P* < 0.005 FWE-corrected] (*B*) Contrast estimates plotted for the reversal trial and the trial before reversal in the vmPFC (for trials with subsequent successful reversal shifts). There are differential neural responses in trials with expected and unexpected outcomes for the distinct trial types (salient loss trials—left; neutral/null trials—right). Avoiding losses under placebo (gray bars) leads to a positive BOLD response in vmPFC in the fully predicted trials and decreased BOLD response in reversal trials signaled by unexpected losses (*P < 0.05; for further statistical reporting see text). This pattern is not seen when individuals reverse their behavior following neutral cues. In contrast, under l-dopa (white bars) we observe the exact opposite pattern, with increased activation for expected outcomes and decreased activation following unexpected outcomes, paradoxically present in null trials and not (as is found under placebo) in the salient trials. Represented are the mean parameter estimates (*β* values). Bars show within-subject standard error.

We first characterized responses under placebo on reversal trials, given that the unexpected outcomes on these trials induce subsequent behavioral reversal shifts. Thus, we tested the contrast (*T*_reversal − 1_− *T*_reversal_) across salient/null blocks in the placebo group, which indexes differences in neural activation for trials with expected outcomes *T*_reversal − 1_ compared with unexpected outcomes cueing reversal *T*_reversal_. Note that these trials differ only in terms of the reversal cue, and that, in both trial types, the sequential behavioral response (Go or No-Go) is identical. Here, we averaged over block (salient/null) and drug condition (levodopa/placebo). This contrast revealed a relative deactivation in vmPFC in response to reversal cues (Fig. 3*A*).

We next tested for the effects of drug (levodopa/placebo) and reversal type (salient/null blocks) on this response to reversal cues, (*T*_reversal − 1_− *T*_reversal_), extracting contrast estimates from a 4-mm sphere centered on the peak coordinate in vmPFC and submitting these to a 2 × 2 ANOVA (reversal type × drug). We observed a significant interaction between drug and reversal type (*F*_1,15_ = 11.73, *P* = 0.004), in the absence of either a main effect of drug (*F*_1,15_ = 0.13, *P* = 0.73) or reversal type salience (*F*_1,15_ = 1,73; *P* = 0.21).

Delineating these effects with post hoc *t*-tests demonstrated the normative pattern on placebo (Fig. 3*B*). In blocks where reversals were signaled by salient monetary losses (i.e., the behavioral goal is to appropriately respond to avoid losses), there was an increase in BOLD signal in vmPFC in trials with fully expected outcomes (i.e., the trial before reversal) and a decreased BOLD response in reversal trials (*t*_(1,14)_ = 2.243, *P* = 0.042). This pattern was not present in the blocks where reversals were signaled by less salient null outcomes. Note this pattern of physiological responses is consistent with the better behavioral performance when subjects were cued to reverse with salient (loss) outcomes.

By contrast, under l-dopa we observed an opposite pattern. Here, there was also an increased activation on the trial before reversal with fully expected outcomes and a decreased activation following reversal cues, but paradoxically this was now present in “null” blocks and not (as is found under placebo) in “salient” blocks (*t*_(1,14)_ = 2.340, *P* = 0.035; Fig. 3*B*). This profile of physiological responses was seen in the context of blunting of the behavioral performance asymmetry between the salient and null blocks under l-dopa, and indeed a partial reversal of this pattern in the behavioral data. These vmPFC responses to expected versus unexpected outcomes were independent of action, with no significant difference in this contrast for “Go” versus “No-Go” blocks, either for placebo or l-dopa [(*T*_reversal − 1_− *T*_reversal_)_Go_− (*T*_reversal − 1_− *T*_reversal_)_No-Go_: placebo *t*_(1,14)_ = 1.09, *P* = 0.292; l-dopa *t*_(1,14)_ = 1.43, *P* = 0.174].

#### Reversal Responses in Insula and Thalamus are Independent of Salience and l-Dopa

**Figure 4.**
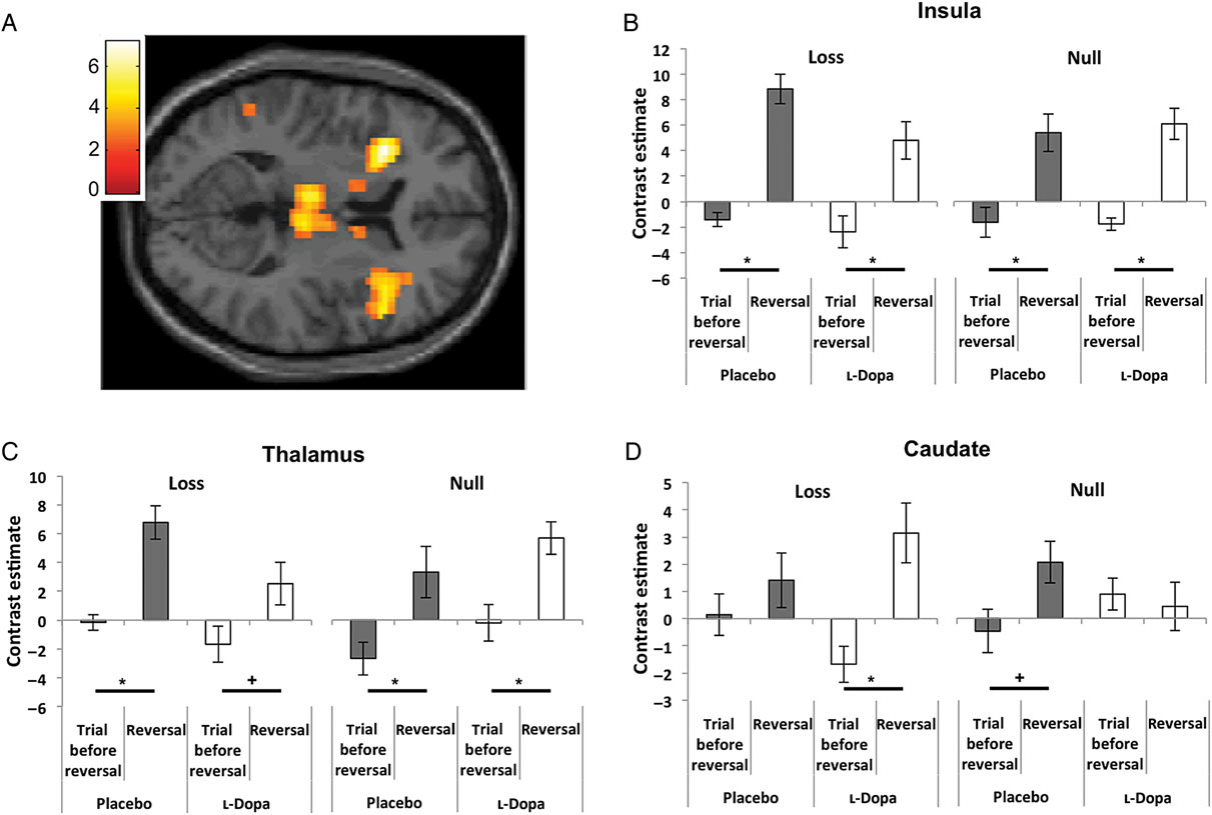
Neurophysiological responses to reversals in caudate, insula, and thalamus. (*A*) Contrast of BOLD activation in a reversal trial (*T*_reversal_, unexpected outcome) minus the trial before reversal (*T*_reversal − 1_, fully expected outcome), averaged over all conditions (placebo/l-dopa and salient/null). Differential activation is seen in bilateral insula, thalamus, and caudate [peak (*x*, *y*, *z*) MNI coordinates (*z* scores)—insula: −27, 17, 7 (4.43) and 42, 20, 1 (5.12); thalamus: −6, −19, 7 (4.74) and 6, −22, 7 (4.08); caudate: 12, 5, 7 (4.37); *P* < 0.005 FWE-corrected]. *(B–D*) Plots of mean parameter estimates (*β* values) for the reversal trial and the trial before reversal in the insula (*B*), thalamus (*C*), and caudate (*D*), contingent upon subsequent successful reversal shifting (i.e., a behaviorally validated response). Here, we observed a prediction error-type response (reduced activation at the time an unexpected/surprising outcome was presented) across trial types [salient loss trials on left; neutral/null trials on right; placebo (gray bars), l-dopa (white bars)]. (*T*_reversal − 1_− *T*_reversal_) contrast effects per condition: bilateral insula – (salient loss)_placebo_: *t*_(1,14)_ = −5.93, *P* < 0.001, (null)_placebo_: *t*_(1,14)_ = −3.41, *P* = 0.004; (salient loss)_l-dopa_: *t*_(1,14)_ = −2.86, *P* = 0.012; (null)_l-dopa_: *t*_(1,14)_ = −5.03, *P* < 0.001. Bilateral thalamus—(salient loss)_placebo_: *t*_(1,14)_ = −4.65, *P* < 0.001, (null)_placebo_: *t*_(1,14)_ = −3.14, *P* = 0.007; (salient loss)_l-dopa_: *t*_(1,14)_ = −1.9, *P* = 0.07; (null)_l-dopa_: *t*_(1,14)_ = −3.11, *P* = 0.008. Left caudate—(null)_placebo_: *t*_(1,14)_ = −1.807, *P* = 0.092; (salient loss)_l-dopa_: *t*_(1,14)_ = −2.803, *P* = 0.014. Within-subject standard error bars are shown. *P < 0.05; ^+^*P* < 0.1.

We next looked for regions with increased activation on reversal trials [i.e., the complementary “prediction error” contrast (*T*_reversal_− *T*_reversal − 1_)]. This highlighted relative increases in activation in caudate, insula, and thalamus (Fig. 4*A*) under both placebo and l-Dopa conditions.

To investigate whether the above-determined regions were also sensitive to reversal type salience or drug effects, we again extracted parameter estimates from 4-mm spheres centered on the peak voxels in these regions and entered the (*T*_reversal_− *T*_reversal − 1_) difference into a 2 × 2 (reversal type × drug) ANOVA. In insula and thalamus, we observed a significant prediction error-type (*T*_reversal_− *T*_reversal − 1_) response in all trial types (Fig. 4*B*,*C*) but no influence of l-dopa or reversal type salience in these regions (insula: *F*_1,15_ = 0.87, *P* = 0.376; thalamus: *F*_1,15_ = 0.449, *P* = 0.514).

#### Caudate Responses to Reversal Cues Depend on Salience

Conversely, there was a significant effect of reversal type salience on the response to unexpected cues within the caudate nucleus—a region implicated in goal directed behavior [(*T*_reversal_− *T*_reversal − 1_) difference: *F*_1,15_ = 7.34, *P* = 0.01]. There were significant increases in BOLD signal on reversal trials (with unexpected outcomes) in the placebo condition (*t*_14_ = 2.17, *P* = 0.048), consistent with a role in signaling prediction error in instrumental tasks (O'Doherty et al. 2004). On l-dopa, we observed a similar pattern of increased BOLD on reversal trials (*t*_(14)_ = 2.68, *P* = 0.018), and also a relative decrease in activation in the trial before reversal (i.e., with fully expected outcomes), but only in blocks where reversals were signaled by salient monetary loss (Fig. 4*D*), and not evident in the blocks where reversals were signaled by null monetary outcomes.

Hence, while we observed complementary responses in vmPFC (decreased responses on reversal trials) and caudate (increased responses on reversal trials), the responses in vmPFC more closely paralleled the observed differences in performance, with more accurate responses on placebo with salient reversal cues, and a similar difference in the profile of neural activation in vmPFC. Significantly, there was a blunting of reversal trial-specific vmPFC responses to salient loss reversal cues following levodopa administration (and a paradoxical enhancement of these responses to null reversal cues).

## Discussion

We characterize the influence of dopamine on set-switching using a paradigm that differs in 2 key ways from typical reversal tasks. In particular, we balanced for the possible effects of dopamine on both movement and motor vigor (requiring both action and action omission), and hedonically salient (negatively valenced) versus nonspecific prediction errors (by employing conditions where reversals were signaled by unpredicted losses or null events). In addition, a deterministic rather than probabilistic reversal contingency allowed us to focus on rapid (inferential) changes in behavioral set rather than gradual acquisition of learned contingencies (in order to minimize reinforcement learning about actions). We found that l-dopa disrupts the normal pattern of response set shifting to salient reversal cues, while preserving overall behavioral accuracy. This was paralleled by obliteration of the normal pattern of prefrontal deactivation in response to salient reversal cues prior to switches in behavior.

A key finding under placebo was that subjects were highly skilled at rapidly altering their behavior following unexpected outcomes, but were significantly less successful at reversal switching when these reversal cues were less salient. Adaptive survival mechanisms, conferred by evolutionary selective pressures, are necessarily tuned to avoidance of salient loss and, in this context, it is unsurprising to find such a striking asymmetry in behavior conditional on the hedonic salience of an outcome. The pattern of behavior under placebo is in stark contrast to what we observed following administration of l-dopa—where an advantage in behavioral switching response to salient cues was lost. This implies that l-dopa degrades the discrimination between a salient loss and a neutral null outcome. This echoes observations that action reprogramming in response to surprising events is dependent on optimal levels of dopamine, and that disorders of dopaminergic transmission can lead to impairments in shifting behavioral set (Berke and Hyman 2000; Hampshire et al. 2012; Galea et al. 2012). Moreover, on l-dopa we observed a trend toward reversal of the normal pattern of behavior, with better reversal switching in response to null outcomes—consistent with evidence that increasing dopamine selectively stabilizes striatal representations of a correct behavioral response for rewards in a Go/No-Go task (Guitart-Masip, Chowdhury et al. 2012) and the observation that dopamine has a differential influence on rewarding compared with punishing outcomes (van der Schaff et al. 2014).

Dopamine has a central role in the maintenance of patterns of neuronal firing in widespread regions of prefrontal cortex (Durstewitz et al. 1999; Seamans and Yang 2004). For example, there is a well-characterized dopaminergic influence on the representational capacity of working memory mediated via an effect on delay-period activity in dorsolateral PFC (Sawaguchi and Goldman-Rakic 1991; Floresco and Phillips 2001; Fuster 2001). More generally, the prefrontal cortex has been suggested to maintain patterns of activity corresponding to behavioral relevant actions and goals (Miller and Cohen 2001). For example, ventrolateral PFC contains neuronal populations coding for stimuli, actions and behavioral context (Asaad et al. 2000). Neurons with task-selective delay-period activity are also present in medial prefrontal cortex (Sakurai and Sugimoto 1986; Baeg et al. 2003), which may underpin the maintenance of appropriate reward-based action–outcome contingencies (Phillips et al. 2004; Deco and Rolls 2005; Floresco et al. 2005; Winter et al. 2009). The vmPFC is a key dopaminergic projection target (Haber 2003) and—along with the DLPFC—is strongly implicated in task switching (Braver et al. 2003; Crone et al. 2006) and behavioral responding to error feedback (Greening et al. 2011). Lesions to medial prefrontal cortex impair reversal switching in both animals (Dias et al. 1996; Schoenbaum et al. 2002) and humans (Fellows and Farah 2003), with structural integrity of the ventromedial and orbitofrontal PFC being crucial for altering behavior in response to negative feedback (Tsuchida et al. 2010).

A current behavioral set is thought to be encoded by a specific pattern of itinerant dynamics that serves to represent a sequence of predicted states, a pattern supported or stabilized by dopamine (Frank et al. 2001; Friston et al. 2012) possibly by modulating the signal-to-noise ratio in specific cortico-striatal circuits (Bamford et al. 2004). In predictive coding accounts of action selection (active inference), dopamine is thought to encode the precision (gain) of prediction errors that induce and maintain a particular response set (Friston et al. 2012; Galea et al. 2012). This latter mechanism predicts that unexpected outcomes should lead to a reduction in dopaminergic modulation of prefrontal cortex, allowing escape from one pattern of neuronal attractor dynamics to an alternative pattern, facilitating a reversal switch (O'Reilly et al. 2002; Deco and Rolls 2005). Compatible with these ideas, we observed in some conditions a decrease in vmPFC BOLD response in reversal trials. This decrease in vmPFC activity at the point of reversal is also consistent with previous observations in operant tasks requiring tracking of a stimulus–response–outcome set (O'Doherty et al. 2003), and perhaps reflect the loss of cue-selectivity within prefrontal neurons, as noted in electrophysiological recordings of reversal tasks (Schoenbaum et al. 2007). Although dorsolateral PFC has a well-established role in maintenance of representations—often in the context of working memory paradigms—interestingly, we did not observe activation in this region. This may well reflect the fact that DLPFC supports more abstract (perceptual) maintenance functions while ventromedial PFC may support maintenance of specific stimulus–outcome contingencies (Ostlund and Balleine 2007; Hampshire et al. 2012). Our paradigm focused upon reward-based (i.e., hedonic) stimulus–outcome associations rather than maintenance of perceptual representations, and we note that there is good evidence in the literature to highlight a role for medial PFC regions in supporting a sustained representation of “expected value,” including in sequential tasks with delayed or deferred outcomes (e.g., Symmonds et al. 2010). In other words, a vmPFC may subserve a role here akin to that of DLPFC, but in the domain of maintenance for stimulus–action–outcome associations that are hedonically relevant.

Significantly, the modulation of vmPFC activity in response to unexpected outcomes in the placebo condition was evident only for salient (negatively valenced) outcomes, paralleling our behavioral findings of better performance in this condition. Moreover, boosting brain dopamine levels by pretreating with l-dopa blunted this vmPFC response to negatively valenced trials, and paradoxically enhanced a response to null events. In other words, the behavioral decrement (in terms of the reduced effect of salience on performance) can potentially be explained by the fact that a relative prefrontal deactivation that occurs in the salient reversal condition on placebo is also seen to occur in the null reversal condition on l-dopa. This strongly indicates that vmPFC is attuned to maintenance of behavioral set contingent on hedonically salient outcomes (i.e., rewarding or punishing) over and above sensitivity to generally surprising outcomes. Dopaminergic disruption selectively obliterates this intrinsic sensitivity to valenced outcomes, although overall behavioral performance is maintained, and this is suggestive that modulation by l-dopa may lead to aberrant attachment of salience to nonsalient outcomes. This is also consistent with findings that vmPFC is involved in the representation of value and reward (O'Doherty et al. 2001; Gottfried et al. 2003) and unites ideas that vmPFC is involved in tracking value (Padoa-Schioppa and Assad 2006) with an hypothesis it is also involved in maintenance and direction of behavioral set (Fellows and Farah 2003; Rushworth et al. 2007).

The PFC has a key role in the control of action (Fuster 2001), while dopamine acts to influence motor responses (Salamone et al. 2003; Guitart-Masip, Chowdhury et al. 2012a). In light of this, it was essential to control for motor execution when identifying a role for dopamine in set shifting. Importantly, the effects seen in vmPFC were invariant to the execution or omission of a motor response. This supports the notion that these neuronal pools encode a behavioral set per se, independent of the commission (execution) of an action, with dopamine enabling maintenance of a representation of specific state-sequences rather than simply facilitating movement.

In healthy individuals, intrinsic dopamine levels are likely to be optimized. Given that the innate salience of negative outcomes is also likely to reflect a well-tuned behavioral adaptation, it is perhaps unsurprising that our dopamine manipulation obliterated this normal sensitivity to loss. It is tempting to speculate that enhancement of dopamine in this experiment has led to degraded task performance on the basis of the well-established suggestion of an inverted U-shaped relationship between dopamine and performance (Cools and D'Esposito 2011; Stuss and Knight 2013). However, we acknowledge here a considerable interindividual variability and as we cannot assay individual dopaminergic function we cannot draw are any conclusions in this regard.

Dynamical (attractor-based) models of response set reversals entail suppression of responses at one level of a neural hierarchy coupled to enhanced responses at complementary levels because of reciprocal information passing (Friston 1997). In these models, a lower level represents or predicts the sequence of actions and outcomes under the current set, and a higher (e.g., prefrontal) level represents the current set itself. Although one could envisage that different levels of such an inferential neuronal hierarchy are instantiated by specialized neuronal pools within prefrontal cortex (Deco and Rolls 2005), the function and architecture of topographically distinct cortico-basal ganglia loops and pathways (Chevalier and Deniau 1990; Haber 2003), and a wealth of behavioral evidence (e.g., Frank and Claus 2006; Cools, Lewis et al. 2006; Wunderlich et al. 2012; Guitart-Masip, Huys et al. 2012) strongly implicates cortico-striatal interactions in directing goal-orientated behavior. Given this integration, we anticipated a reciprocity of response patterns (i.e., relative activations at one level of a hierarchy coupled to relative deactivations at an adjacent level). We observed a degree of reciprocity in response to reversal cues, with decreases in vmPFC responses concurrent with increases in caudate responses. This echoes a neurochemical reciprocity between the PFC and striatum, whereby increases in PFC dopamine are associated with decreases in the basal ganglia and vice versa (Pycock et al. 1980; van Schouwenburg et al. 2010). This may partly reflect caudate involvement in signaling prediction error during instrumental learning tasks (O'Doherty 2004; Clarke et al. 2011). It also accords with theories suggesting a “gating” role for the basal ganglia (Frank and Claus 2006; McNab and Klingberg 2008). The strongest established prefrontal-striatal anatomical connections are between caudate and lateral prefrontal areas. However, the vmPFC also projects to the medial wall of the caudate nucleus, adjacent to the ventricle (Haber and Knutson 2009). Moreover, striatal projection fields from medial prefrontal cortex extend throughout central and dorsal caudate and putamen nuclei, and there is substantial cross-talk between cortico-striatal circuits at the level of the striatum (Kasanetz et al. 2008). It is therefore possible that the caudate region we observed directly reflects this anatomical reciprocity between cortex and striatal hierarchical levels. It is also probable that the caudate plays a role in supporting behavioral performance in this set-shifting task. We note that the caudate is implicated in goal directed value representation (Wunderlich et al. 2012). We suggest that the difference in caudate activation under l-dopa is likely to contribute to the observed differences in behavioral accuracy. However, it is also important to note that prefrontal responses to reversals in the normative (placebo) condition were most marked following salient loss outcomes in a way not seen within striatal regions.

It is interesting to note that we also observed “prediction error”-type responses in thalamus and insula, with increased activation following unexpected cues during reversals. The insula, which enjoys reciprocal connections to striatum, thalamus and frontal cortex (Flynn 1999), has been associated with interoception, salience, monitoring uncertainty, and tracking emotionally relevant events (Craig 2003; Critchley et al. 2004; Preuschoff et al. 2008; Cauda et al. 2012). The medial thalamus also projects to the vmPFC (Derrfuss et al. 2004) and striatum (Vertes et al. 2012). In a hierarchical model structure, one speculation is that these regions may represent predictions about interoceptive states (Gu et al. 2013) and lower level sensations (Adams et al. 2013), respectively. The influence of several regions expressing prediction error-type responses could drive response set-switching for hedonically “null” outcomes, and explain why overall accuracy is maintained on l-dopa despite the alterations seen in prefrontal cortical activation.

More broadly, it has been suggested that PFC and basal ganglia dopamine regulate a balance between 2 functionally opponent processes, with PFC dopamine regulating behavioral stability and the basal ganglia dopamine promoting flexibility (van Schouwenburg et al. 2010). Our data enrich these theories by suggesting that (metastable) representations of behavioral sets are maintained in sectors of PFC under a dopaminergic influence, and that the breakdown and re-establishment of cortical dynamics—corresponding to a reversal switch—relies on transient suppression of this influence, following an unexpected outcome. In turn, this suppression is disrupted by exogenous l-dopa administration, with evidence that vmPFC in particular is driving the observed behavioral differences. This resonates with a previous demonstration of dopamine-induced changes in striatal functional connectivity, whereby l-dopa simultaneously increases functional connectivity between striatum and motor outflow regions in brainstem and cerebellum, while reducing (functional) connectivity between striatum and medial prefrontal cortex (Kelly et al. 2009).

In conclusion, we show an effect of salience on set-switching, with more efficient reversal following unexpected losses compared with null outcomes was abolished by l-dopa, both at the level of behavior and associated prefrontal responses. Context sensitive processing requires an intact interplay between various cortical and subcortical regions that form the brains reward circuits which is impacted by alteration of endogenous dopamine levels in the brain (Diekhof et al. 2008). This alteration in a balance between the impact of hedonically salient, compared with neutral, outcomes might underlie a range of phenomena, including the bias in salience attribution to cues observed in the depressive state, addiction (Diekhof et al. 2008) and Tourette syndrome (Gilbert et al. 2006) all of which implicate dysfunction in dopaminergic pathways. It might also explain common clinical syndromes such as pathological gambling, where there is a lack of avoidance of negatively reinforced actions and impulse control disorders in Parkinson's disease (Evans et al. 2004), where a blunting or reversal of the normal suppression of activity might explain a paradoxical maintenance of behavioral set in the face of negative outcomes.

## Funding

This work was supported by the Wellcome Trust (Ray Dolan Senior Investigator Award 098362/Z/12/Z); The Wellcome Trust Centre for Neuroimaging is supported by core funding from the Wellcome Trust (091593/Z/10/Z). T.S. was an MRC Clinical Research Training Fellow. Funding to pay the Open Access publication charges for this article was provided by the Wellcome Trust.

## Notes

*Conflict of Interest*: None declared.
